# Simple Modification of the Bladder Outlet Obstruction Index for Better Prediction of Endoscopically-Proven Prostatic Obstruction: A Preliminary Study

**DOI:** 10.1371/journal.pone.0141745

**Published:** 2015-10-27

**Authors:** Jang Hee Han, Ho Song Yu, Joo Yong Lee, Joohan Kim, Dong Hyuk Kang, Jong Kyu Kwon, Young Deuk Choi, Kang Su Cho

**Affiliations:** 1 Department of Urology, Gangnam Severance Hospital, Urological Science Institute, Yonsei University College of Medicine, Seoul, Korea; 2 Department of Urology, Chonnam National University Medical School, Gwangju, Korea; 3 Department of Urology, Severance Hospital, Urological Science Institute, Yonsei University College of Medicine, Seoul, Korea; 4 Department of Mechanical Engineering, Seoul National University of Science & Technology, Seoul, Korea; 5 Department of Urology, Yangpyeong Health Center, Yangpyeong, Korea; Cedars-Sinai Medical Center, UNITED STATES

## Abstract

**Purpose:**

The bladder outlet obstruction index (BOOI), also known as the Abrams-Griffiths (AG) number, is the most widely used index for predicting BOO. However, the obstructed prostatic urethra determined by the BOOI is often inconsistent with endoscopically-proven obstruction. We assessed abdominal straining pattern as a novel parameter for improving the prediction of BOO.

**Materials and Methods:**

We retrospectively reviewed the pressure-flow studies (PFS) and cystourethroscopy in 176 BPH/LUTS patients who were unresponsive to medical therapy. During PFS, some groups of patients tried to urinate with abdominal straining, which can increases intravesical pressure and underestimate BOOI theoretically. Accordingly, the modified BOOI was defined as (P_det_Q_max_+ΔP_abd_)-2Q_max_.

**Results:**

Ultimately, 130 patients were eligible for the analysis. In PFS, ΔP_abd_ (P_abd_Q_max_-initial P_abd_) was 11.81±13.04 cmH_2_O, and it was 0–9 cmH_2_O in 75 (57.7%), 10–19 cmH_2_O in 23 (17.7%) and ≥20 cmH_2_O in 32 (24.6%) patients. An endoscopically obstructed prostatic urethra in 92 patients was correctly determined in 47 patients (51.1%) by the original BOOI versus 72 patients (78.3%) based on the modified BOOI. Meanwhile, an “unobstructed” urethra according to the original BOOI was present in 11 patients (12.0%), whereas according to the modified BOOI, only 2 (2.1%) would be labeled as “unobstructed”. In receiver operating characteristic curves, the area under the curve was 0.906 using the modified BOOI number versus 0.849 in the original BOOI (p<0.05).

**Conclusions:**

The change in abdominal pressure was correlated with endoscopically-proven obstruction. Our simple modification of the BOOI on the basis of this finding better predicted bladder outlet obstruction and, therefore, should be considered when evaluating BOO in patients with LUTS/BPH.

## Introduction

Benign prostatic hyperplasia (BPH), which includes benign prostatic enlargement (BPE) and benign prostatic obstruction (BPO), has conventionally been considered a major factor in causing male lower urinary tract symptoms (LUTS) [1]. Recently, the pathophysiology of male LUTS has been regarded as being highly complex and multifactorial [2]. Accordingly, identifying the existence of bladder outlet obstruction (BOO) in patients with LUTS is important, especially when considering an invasive treatment in the management of medically intractable patients. Some reports have demonstrated that the correlation between the absolute prostate volume and LUTS severity is weak, and prostate volume itself is also not directly correlated with BPO or BOO [3,4]. Therefore, framing a nomogram to accurately predict BOO is an important issue [5,6].

Urodynamic studies, especially pressure-flow studies (PFS), are known to be the gold standard tool for diagnosing the existence of BOO. Since 1972, studies have been attempting to simplify the diagnosis of BOO in men and to create a standardized method for diagnosis using PFS. The best-known methods for the diagnosis of BOO are the Abrams-Griffiths (AG) nomogram and number, the Schafer nomogram, the group-specific urethral resistance factor (URA), the detrusor-adjusted mean passive urethral resistance relation (PURR), and the obstruction coefficient (OCO) [1].

**Fig 1:**
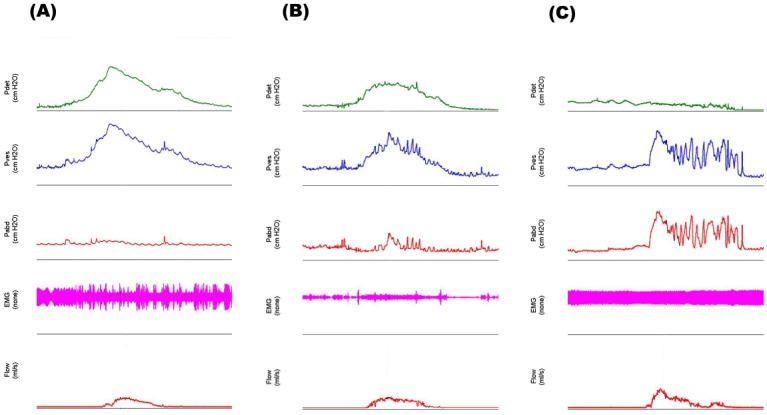
Different patterns of abdominal pressure (p_abd_) and detrusor pressure (p_det_) during the pressure-flow studies (PFSs). (A) Patient voids using detrusor pressure with no meaningful abdominal pressure. (B) Patient voids mainly using detrusor pressure but with the addition of abdominal pressure. (C) Patient voids predominantly by abdominal pressure (abdominal straining). EMG = electromylography, p_ves_ = vesical pressure.

The AG nomogram is a well-established and widely-adopted method to diagnose the presence of BOO [7,8]. The AG number (also known as the BOO index [BOOI]) is derived from the equation for the slope of the line dividing the obstructed from the equivocal in the AG nomogram, which is the most simple and practical way in combined of the P_det_Q_max_ and Q_max_ [8]. However, quite a large portion of patients try to urinate with abdominal straining during a PFS, which can increase intravesical pressure, and subsequently increase Q_max_ (Fig 1). In other words, Q_max_ is also affected by the abdominal pressure (straining) as well as by the detrusor pressure. From the viewpoint of fluid dynamics, it might be more reasonable to consider the increased intravesical pressure in addition to P_det_Q_max_ when determining BOO on the PFS. In the current study, we assessed abdominal pressure as an independent factor in predicting BOOI and consequently investigated whether the modification of the AG number when considering the additional change of abdominal pressure could enhance the diagnostic accuracy of PFS.

## Materials and Methods

### Patients

Medical records and our urodynamic study database between Mar 2010 and Jan 2014 were retrospectively reviewed. We carefully searched 170 LUTS/BPH patients who underwent both PFS and cystourethroscopy due to unresponsiveness to medical therapy and who were being considered for invasive therapy. Unresponsiveness to medical therapy was defined when the urinary symptom score and the urinary flow rate did not improve, despite the administration of alpha blocker with or without 5a reductase–inhibitor treatment for more than 6 months. Men with prostate and bladder cancer, radiotherapy of the pelvis, urethral stricture, urethral stones, history of prostatectomy, and any evidence of neurological disease that could affect voiding function were initially excluded. A total of 158 men were initially eligible for our study. Of the eligible men, 28 patients failed to void during the PFS, therefore, the data from 130 patients were included in the final analysis.

### Good Clinical Practice Protocols

The study was performed in accordance with applicable laws and regulations, good clinical practices, and the ethical principles described in the Declaration of Helsinki. The Institutional Review Board of Severance Hospital approved this study protocol (Approval No. 4-2014-0835). The need for written informed consent given by participants was exempted due to the retrospective study design, and patient records and information was anonymized and de-identified prior to analysis.

### Assessment of Urinary Symptoms and Uroflowmetric Measurements

LUTS were evaluated using the International Prostate Symptom Score (IPSS) and the Overactive Bladder Symptom Score (OABSS). The total IPSS was subcategorized into the voiding, storage, and post micturition symptom scores. For uroflowmetric measurements, peak urinary flow rate (Q_max_) and post-void residual volume (PVR) was evaluated. Both uroflowmetric and PVR measurements were repeated if the voided volume was less than 125 mL. The assessment was made using the Bluetooth uroflowmetry (Urodyn+; Mediwatch UK, Ltd., Rugby, United Kingdom) and a bladder scanner (BioCon-500; MCube Technology Co. Ltd., Seoul, Korea).

### Urodynamic and Cystourethroscopic Evaluation

Multichannel urodynamic evaluation (Laborie Aquarius TT urodynamic equipment—Laborie Medical Technologies, Mississauga, ON, Canada) was performed using the methods and definitions conforming to those of the International Continence Society [9]. A filling cystometry and PFS were conducted with the patient in a sitting position, with external pressure transducers, a 6-French double-lumen transurethral catheter to measure the intravesical pressure, and a 10-French single-lumen rectal balloon catheter to measure the intra-abdominal pressure. Sterile physiological saline solution was infused through the transurethral catheter at an infusion speed of 30–50 ml/min.

Four methods were used to assess bladder outlet obstruction: (1) BOOI = P_det_Q_max_-2Q_max_ [10], (2) modified BOOI = P_det_Q_max_+ΔP_abd_-2Q_max_, (3) urethral resistance factor (URA) = ([1+1.52*10^-3^*P_det_Q_max_^2^]^1/2^-1)/(7.6*10^-4^*Q_max_^2^) [11,12], and (4) the obstruction coefficient (OCO) = P_det_Q_max_/(40+2Q_max_) [13]. BOOI was designed by Abrams and Griffiths in 1979. Under empiric observations and theoretical considerations, pressure-flow plotting was divided into three zones and analyzed. Men are considered obstructed if BOOI is >40, unobstructed if BOO is <20, and equivocal if BOOI is between 20 and 40. Griffiths invented the concept of URA, the urethral opening pressure, in 1989. In this formula, the detrusor pressure and urinary flow rate were designed as components in the quadric equation, introducing the concept of compressive obstruction and constrictive obstruction [13]. The concept of OCO was introduced by Schaefer in 1995. OCO combined the maximum flow rate and maximum flow pressure into a single numeric factor, which evaluated the relative deviation from the obstruction line in the Abrams-Griffiths nomogram [14]. The cutoff value drawn from our study was compared with the conventional diagnostic cutoff for OCO (>1), URA (>29), and BOOI (>40).

Cystourethroscopy was performed by a single urologist (K.S.C.), and a 20 Fr rigid cystoscopic sheath was used. The prostatic urethra was inspected at the veru montanum while saline was continuously infused at the pressure of 40 cmH_2_O. An endoscopically obstructed prostatic urethra was defined when both lobes of the prostate were completely kissing, and regarded as the reference standard, concordant with “severe obstruction” suggested by el Din et al [15]. Two investigators (J.H.H. and H.S.Y.) reviewed the endoscopic images and determined the prostatic urethral obstruction independently. Disagreement between the two investigators was resolved by a discussion with another investigator (K.S.C.).

### Statistical Analyses

Variables in the two groups were compared using Student’s t-test, the Mann-Whitney U test, and the chi-square test. In multivariable models, a backward stepwise elimination process was carried out to identify the most relevant measurements of urodynamic parameters for predicting BOO. This process started with a full model containing all candidate measurements. By stepwise deletion of non-significant variables according to the likelihood-ratio criteria, the final multivariable model contained only significant variables. Based on the result above, multivariate logistic regression analysis was performed with three parameters: ΔP_abd_ and the two conventional parameters PdetQmax and Qmax, which were used in all four methods. Receiver operating characteristic (ROC) curves were calculated to evaluate the positive impact of the abdominal pressure parameter.

Four method’s grouping results were compared to the endoscopic finding, and the sensitivity, specificity, positive predictive value (PPV), negative predictive value (NPV), and the diagnostic accuracy of each method were calculated. Consequently, the diagnostic accuracy and the optimal cutoff value point of each method were analyzed by a receiver operating characteristic (ROC) curve. The area under the curve (AUC) for each methods were calculated, and compared using an algorithm suggested by DeLong et al [16]. The cutoff values for each parameter (BOOI, modified BOOI, URA, and OCO) were estimated to where the value corresponds to the highest accuracy. A decision curve analysis was performed to determine whether the modified BOOI model increased the net benefit over a realistic range of thresholds when compared with other models.

All statistical analyses were performed using the R statistical software (R version 3.0.2, R Foundation for Statistical Computing, Vienna, Austria; http://www.r-project.org). A P-value <0.05 was considered statistically significant, and all statistical tests were two-sided.

## Results

On cystourethroscopic evaluation, 92 patients (70.8%) were diagnosed as “endoscopically obstructed”, and 38 patients (29.2%) as “endoscopically unobstructed”. As for the urinary symptom questionnaires, the obstructed group had more severe symptom scores in IPSS-total, IPSS-storage, and OABSS. However, there was no difference in IPSS-voiding and IPSS-post-micturition. The transrectal ultrasonographic examination revealed that the prostate volume, transitional zone volume, and transitional zone index in the obstructed group was significantly higher. On the free-flow study, Q_max_ was lower in the obstructed group, but there was no difference in the post-void residual between the two groups (Table 1).

**Table 1:** Clinical features of subjects according to endoscopic finding of prostatic urethra.

	Endoscopic finding of prostatic urethra		P-value
	Unobstructed	Obstructed	
**Number (%)**	**38 (29.2)**	**92 (70.8)**	
**Age (year)**	**66.95 ± 8.17**	**71.02 ± 8.18**	**0.011**
**Hypertension (%)**	**12 (31.6)**	**42 (45.7)**	**0.139**
**Diabetes mellitus (%)**	**11 (28.9)**	**21 (22.8)**	**0.461**
**Urinary symptom questionnaire**			
** IPSS, total**	**18.39 ± 6.38**	**21.64 ± 7.45**	**0.034**
** IPSS, storage**	**7.10 ± 2.70**	**8.55 ± 3.78**	**0.026**
** IPSS, voiding**	**9.13 ± 3.78**	**9.39 ± 3.93**	**0.751**
** IPSS, post-micturitional**	**2.53 ± 1.59**	**3.15 ± 1.62**	**0.069**
** OABSS**	**5.15 ± 2.60**	**6.91 ± 3.79**	**0.028**
**Transrectal ultrasonography**			
** Prostate volume**	**29.06 ± 10.55**	**50.89 ± 21.20**	**<0.001**
** Transitional zone volume**	**13.92 ± 6.16**	**29.16 ± 16.63**	**<0.001**
** Transitional zone index**	**0.47 ± 0.10**	**0.54 ± 0.14**	**0.009**
**Uroflowmetry (free-flow study)**			
** Q**_**max**_ **(mL/s)**	**10.09 ± 5.32**	**8.15 ± 4.12**	**0.003**
** Post void residual (mL)**	**59.29 ± 57.48**	**75.77 ± 80.22**	**0.274**

IPSS: International Prostate Symptom Score, OABSS: Overactive Bladder Symptom Score

**Fig 2:**
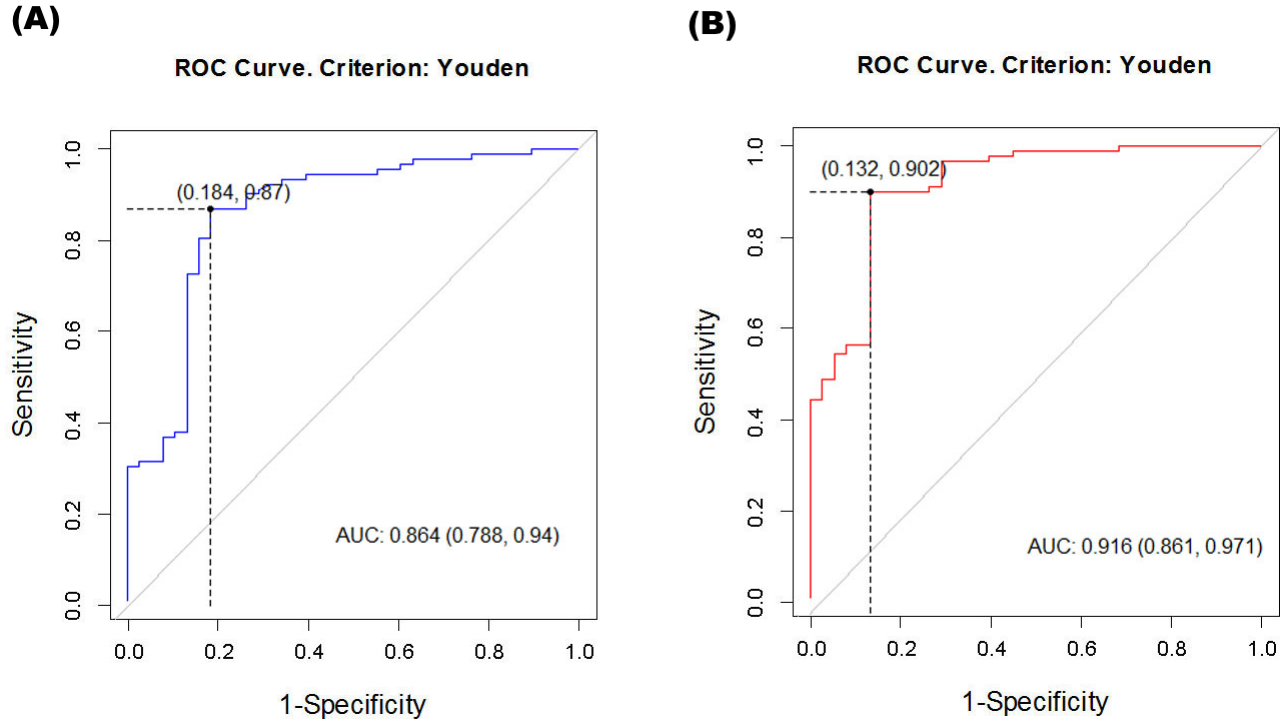
Receiver operating characteristic (ROC) curves of two models extracted from multivariate logistic regression. (A) PdetQmax, Qmax (B) PdetQmax, Qmax, ΔPabd.

In the filling cystometry study, the volume at first desire, the volume at urgency, and maximum cystometric capacity were significantly smaller in the obstructed group, and compliance was also lower in the obstructed group. During the PFS, there was no difference in Q_max_ between the two groups, but P_det_Q_max_ was 32.66 ± 15.22 cmH_2_0 in the unobstructed group versus 61.22 ± 27.13 cmH_2_0 in the obstructed group (P < 0.001). Notably, ΔP_abd_ was significantly higher in the obstructed group than in the unobstructed group (9.0 [3, 23.75] cmH_2_O vs. 3.50 [0, 10] cmH_2_O; P = 0.001). Subjects with a ΔP_abd_ ≥10 cmH_2_O composed 48.9% of the obstructed group but only 26.3% of the unobstructed group (Table 2). The ROC curve for ΔP_abd_ was calculated and the AUC was 0.679 (not shown in figures). We compared the two ROC curves using the probability variables extracted from the two independent multivariate logistic regression models: (A) PdetQmax and Qmax and (B) PdetQmax, Qmax, and ΔP_abd_. The latter model was highly predictive of endoscopically proven obstruction (AUC = 0.916; p < 0.001). The former model with the two conventional variables had an ROC curve with an AUC of 0.864 (p < 0.001) Comparison of the two methods was performed, and the former model was superior, with a p-value of 0.0058 (CI: 0.0171–0.101; Fig 2).

**Table 2:** Summary of multichannel urodynamic study according to endoscopic finding of prostatic urethra.

	Endoscopic finding of prostatic urethra		P-value
	Unobstructed (N = 38)	Obstructed (N = 92)	
**Filling cystometry**			
** First desire (mL)**	**276.95 ± 106.99**	**226.50 ± 97.96**	**0.010**
** Urgency (mL)**	**448.95 ± 168.26**	**372.61 ± 149.71**	**0.012**
** MCC (mL)**	**474.11 ± 180.77**	**385.76 ± 154.15**	**0.006**
** P**_**det**_ **at MCC (cmH**_**2**_ **O)**	**9.34 ± 6.86**	**11.96 ± 13.47**	**0.258**
** Compliance (mL/cmH**_**2**_ **O)**	**80.47 ± 66.18**	**55.66 ± 46.29**	**0.016**
** Detrusor overactivity (%)**	**10 (26.3)**	**43 (46.7)**	**0.031**
**Pressure-flow study**			
** Q**_**max**_ **(mL/s)**	**9.87 ± 5.97**	**8.04 ± 4.58**	**0.062**
** P**_**det**_ **Q**_**max**_ **(cmH**_**2**_ **O)**	**32.66 ± 15.22**	**61.22 ± 27.13**	**<0.001**
** ΔP**_**abd**_ **(cmH**_**2**_ **O)**	**3.50 [0, 10]**	**9.0 [3, 23.75]**	**0.001** *
** <10 (%)**	**28 (73.7)**	**47 (51.1)**	
** ≥10, <20 (%)**	**6 (15.8)**	**17 (18.5)**	
** ≥ 20 (%)**	**4 (10.5)**	**28 (30.4)**	
**BOOI**	**12.89 ± 19.44**	**45.11 ± 28.55**	**<0.001**
**Modified BOOI**	**19.53 ± 16.98**	**59.05 ± 28.83**	**<0.001**
**URA**	**20.03 ± 9.91**	**35.84 ± 17.20**	**<0.001**
**OCO**	**0.57 ± 0.29**	**1.12 ± 0.53**	**<0.001**

MCC: Maximum cystometric capacity, BOOI: bladder outlet obstruction index, URA: urethral resistance factor, OCO: obstruction coefficient. Independent t-tests and chi-square tests were applied for continuous and categorical variables.

*p-value: Mann-Whitney U test was applied

**Fig 3:**
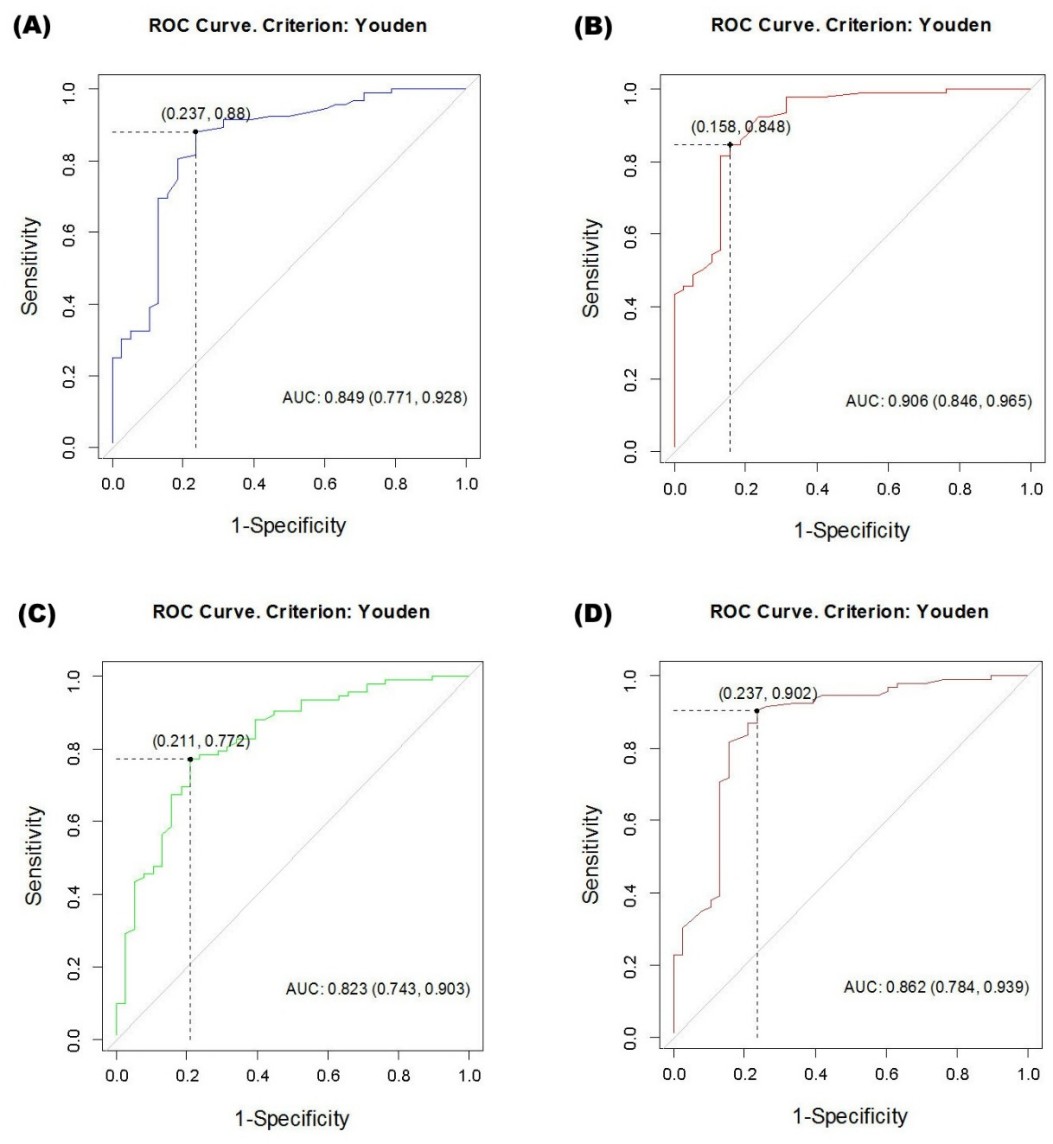
Receiver operating characteristic (ROC) curves of four different methods to predict bladder outlet obstruction (BOO). (A) Bladder outlet obstruction index (BOOI), (B) Modified bladder outlet obstruction index (modified BOOI), (C) Urethral resistance factor (URA), (D) Obstruction coefficient (OCO). AUC = Area under the curve.

**Fig 4:**
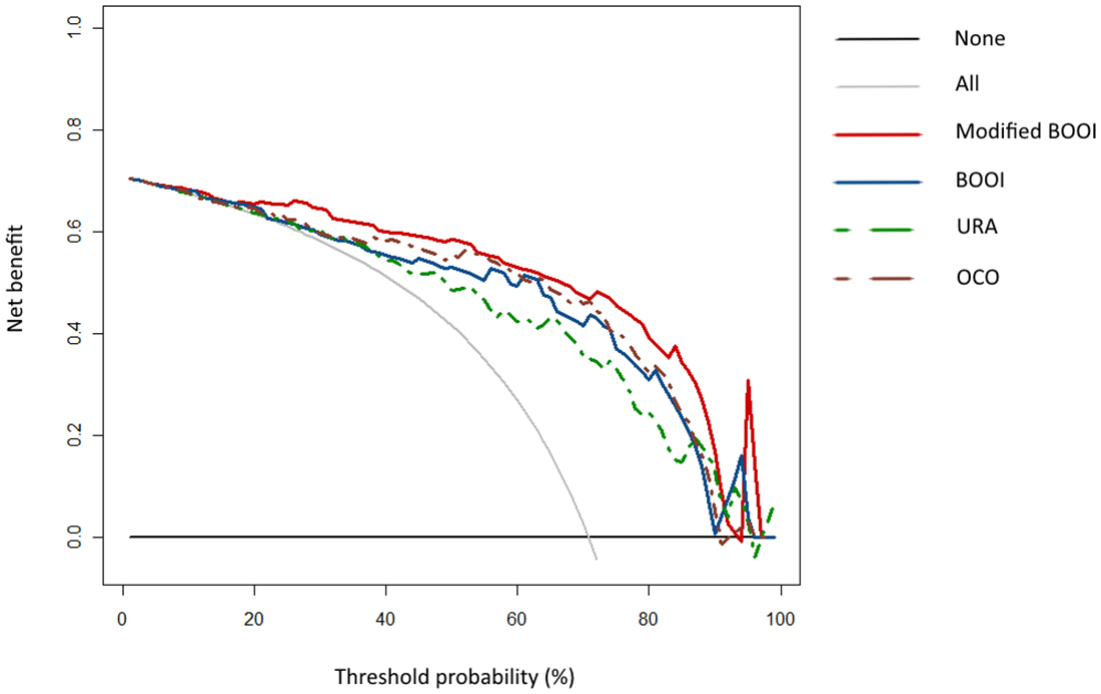
Decision curve analysis (DCA) of four different methods for predicting bladder outlet obstruction (BOO).

Among 92 patients showing endoscopic prostatic obstruction, only 47 patients (51.1%) were determined as “obstructed” according to the original BOOI, but 72 patients (78.3%) were classified as “obstructed” based on the modified BOOI. Additionally, an “unobstructed” urethra according to the original BOOI was present in 11 patients (12.0%), whereas according to the modified BOOI, only 2 (2.1%) would be labeled as “unobstructed”. Diagnostic test validation was performed, and for the 2×2 table validation, the “equivocal group” was classified as the unobstructed group. Validation of each of the methods was based on the accepted cutoff values: OCO (>1), URA (>29), BOOI (>40), and modified BOOI (>40). Of the four methods compared, including BOOI, modified BOOI, URA, and OCO, the modified BOOI showed the highest values in all parameters for test validity (PPV = 0.94, NPV = 0.62, sensitivity = 0.78, specificity = 0.87, and diagnostic accuracy = 0.81; Table 3). To verify and compare the validity of the four methods for the diagnosis of endoscopic prostatic obstruction, ROCs were also generated. This method demonstrated that the AUC was 0.906 in the modified BOOI, 0.849 in BOOI, 0.823 in URA, and 0.862 in OCO. By using pairwise comparison of ROC curves according to the DeLong algorithm, the diagnostic accuracy of the modified BOOI was proven to be significantly better than BOOI (p = 0.0016), OCO (p = 0.014), and the URA (p = 0.0001). The optimal cutoff values of each method were 33.0 in the modified BOOI, 20.0 in BOOI, 24.3 in URA, and 0.62 in OCO (Fig 3). The decision curve analysis showed that adding ΔP_abd_ to the BOOI was superior to the original BOOI, as well as the two other methods, with greater net benefit across a wide range of risks (Fig 4).

**Table 3:** Diagnostic accuracy of four methods for the evaluation of bladder outlet obstruction.

	Endoscopic finding of prostatic urethra		Total (N = 130)
	Unobstructed (N = 38)	Obstructed (N = 92)	
***BOOI***			
**Unobstructed (%)**	**29 (76.3)**	**11 (12.0)**	**40 (30.8)**
**Equivocal (%)**	**4 (10.5)**	**34 (36.9)**	**38 (29.2)**
**Obstructed (%)**	**5 (13.2)**	**47 (51.1)**	**52 (40.0)**
**PPV = 0.90, NPV = 0.42, Sensitivity = 0.51, Specificity = 0.87, Accuracy = 0.62**			
***Modified BOOI***			
**Unobstructed (%)**	**26 (68.4)**	**2 (2.1)**	**28 (21.6)**
**Equivocal (%)**	**7 (18.4)**	**18 (19.6)**	**25 (19.2)**
**Obstructed (%)**	**5 (13.2)**	**72 (78.3)**	**77 (59.2)**
**PPV = 0.94, NPV = 0.62, Sensitivity = 0.78, Specificity = 0.87, Accuracy = 0.81**			
***URA***			
**Unobstructed (%)**	**23 (60.5)**	**16 (17.4)**	**39 (30.0)**
**Equivocal (%)**	**9 (23.7)**	**21 (22.8)**	**30 (23.1)**
**Obstructed (%)**	**6 (15.8)**	**55 (59.8)**	**61 (56.9)**
**PPV = 0.90, NPV = 0.46, Sensitivity = 0.60, Specificity = 0.84, Accuracy = 0.67**			
***OCO***			
**Unobstructed (%)**	**33 (86.8)**	**43 (46.7)**	**76 (58.5)**
**Obstructed (%)**	**5 (13.2)**	**49 (53.3)**	**54 (41.5)**
**PPV = 0.91, NPV = 0.43, Sensitivity = 0.53, Specificity = 0.87, Accuracy = 0.63**			

BOOI: bladder outlet obstruction index, URA: urethral resistance factor, OCO: obstruction coefficient, PPV: positive predictive value, NPV: negative predictive value

## Discussion

Uroflowmetry and PVR are simple tests that are performed when BOO is suspected. However, a decreased maximum flow rate can result from either an impaired detrusor contractility or BOO [17,18]; therefore, Q_max_ is unable to distinguish groups with impaired detrusor contractility from BOO without a synchronous measurement of detrusor pressure (P_det_). Indeed, 25–30% of men with decreased flow were not obstructed in the Abrams’ study [19].

Due to the weakness of uroflowmetry, the PFS has been used for better definition and grading of the degree of BOO. Several nomograms using a PFS have been introduced to evaluate BOO, and researchers have compared the diagnostic accuracy of the nomograms in various ways. Jimao et al. compared the degree of the agreement between the AG nomogram, URA, and detrusor-adjusted mean passive urethral resistance relation factor (DAMPF); the authors showed the cutoff value in the ROC curve of each method [1]. Cravalho et al. compared the agreement in obstruction measurements by the AG nomogram, passive urethral resistance relation (PURR), and the micturitional urethral pressure profile (MUPP) [20]. After reviewing the study results, the current consensus is to use BOOI as a representative method due to its simplicity and similar diagnostic accuracy.

Three methods referred to in the current study (BOOI, URA, and OCO) all used PdetQmax and Qmax as urodynamic parameters to predict BOO. In our study, we compared all urodynamic parameters between the obstructed and unobstructed groups. Of all the parameters, ΔP_abd_ was the second most powerful factor, followed by PdetQmax, as revealed in multivariate logistic regression. Based on intuition and our experience in clinical practice, we hypothesized that ΔP_abd_ could be an important clinical factor and may play a role as an independent factor in raising diagnostic accuracy. We attempted to design a new method that would be simple and easily applicable in the clinical practice, while maintaining the basic frame of the conventional methods, which used PdetQmax and Qmax. Considering the availability of parameters and ease of measurability in the clinical setting, we created the new method simply adding the abdominal pressure parameter to the conventional BOOI method. The purpose of this process was not to design the best predictive model by using three parameters, but merely to investigate the feasibility of applying this method to clinical practice in order to obtain better predictive power.

In the previous studies, there was an absence of clear criteria for endoscopic bladder outlet obstruction. We referred to the diagram shown by el Din et al. for defining and grading BOO [15]. The authors suggested that prostatic occlusion could be classified into three grades: grade 1 (no obstruction), grade 2 (moderate obstruction), and grade 3 (severe obstruction). We defined obstruction as the prostate’s complete kissing (grade 3) with a hydrodynamic pressure of 40 cmH_2_O endoscopically. Because we know the real obstruction grade, we could compare the nomograms more precisely and measure the diagnostic accuracy of each method. After the ROC curve was drawn, the AUC of the modified BOOI had the largest AUC (0.906), indicating high accuracy, while the other methods were between 0.8–0.9 (moderate accuracy). With regards to hydromechanics, theoretically, our modified BOOI can supplement the original BOOI to identify the group with low contractility and low flow rate, which voids using the abdominal pressure. The basis of our theory is similar to the penile cuff test, a well-known noninvasive method for predicting BOO. In the penile cuff test, the degree of obstruction is evaluated by measuring the urine flow rate and the pressure of the urethra, which indicates isovolumetric bladder pressure [21]. That test is similar to our modified BOOI in that both take the abdominal pressure into account. Studies have revealed that a patient diagnosed as obstructed following a penile cuff test can be reassured that surgery has a high chance of bringing the symptomatic relief. On the other hand, patients who are diagnosed as unobstructed may wish to try other non-surgical treatment options in light of the likely poor surgical outcome [22].

Predicting who would benefit from invasive treatment modalities is crucial in clinical practice, however, BOOI has several limitations in that respect. Men with a relatively low detrusor pressure at maximum flow and relatively low maximum flow have a high prevalence of discrepancy with URA and Schafer’s grade classifications [12]. In some previous reports, >60% of patients with unobstructed BOOI and weak contractility received benefit from an operation [23,24]. According to another report, a high percentage (70%) of equivocal or unobstructed patients experienced reductions in symptoms compared with the obstructed patients [25]. The authors attribute this result to the removal of the prostatic obstruction that was undetected during the PFS and was regarded as unobstructed in the AG nomogram [23]. Moreover, with regards to the medical treatment, alpha blockers have been proven effective for those who are considered equivocal or unobstructed, as well as for the obstructed group [26,27]. Consequently, a nomogram with greater accuracy in predicting BOO is needed for choosing the correct treatment options.

According to Sekido, P_abd_ significantly contributes to voiding, acting as a compensatory mechanism for patients with a hypo- or acontractile neurogenic bladder. Although the valid method of analyzing voiding with abdominal straining on the PFS remains undecided, Sekido insists that clinicians should carefully observe the raw pressure-flow traces, in addition to using the nomograms [25]. In clinical practice, LUTS/BPH patients also have weak detrusor contractility, and some of those patients urinate with dominant abdominal pressure, as shown in Fig 1. Our modified BOOI could serve a significant role in distinguishing these patients from the obstruction group and could suggest a more accurate indication for performing invasive treatment. Because the approach is a simple modification of the AG number, the quantitative value of modified BOOI might also be used for defining the severity of the obstruction and the efficacy of therapy.

Meanwhile, applying the alternative methods to our modified BOOI can further improve its diagnostic accuracy. Lim et al. demonstrated that equivocal cases grouped by BOOI can be classified into obstructed and unobstructed groups using the pressure-flow slope and minimal voiding detrusor pressure (p_muo_) [8]. If the slope is >2 cmH_2_O per milliliter per second or the p_muo_ is >40 cmH_2_O, then the PFS is obstructed. On the other hand, if the slope is <2 cmH_2_O per milliliter per second or the p_muo_ is <40 cmH_2_O, then the PFS is unobstructed. In our study, among the 92 patients who were endoscopically obstructed, 18 (19.6%) patients were classified as equivocal in the modified BOOI. Among those 18 patients, 5 patients were additionally grouped as “obstructed”, considering p_muo_ and the pressure-flow slope, and elevating its diagnostic accuracy.

Our study had several limitations. We considered the endoscopic prostatic complete kissing as the definition for bladder outlet obstruction. However, in clinical practice, prostatic flow is the result of a variety of influential factors. Flow is not in simple correlation with an endoscopically-proven mechanical obstruction. There are also inherent limitations from the nature of a retrospective study and cross-sectional analysis. Accordingly, a larger prospective cohort study is needed to compare the difference between the preoperative and postoperative PFSs in each patient, in order to confirm the diagnostic usefulness of our modified BOOI. Despite the shortage of the study, this study was maximally controlled in the process of evaluation, and fully demonstrated the possibility of a potentially competitive nomogram. In view of the current data, future studies, which consider the differences in abdominal pressure as a significant clinical factor in predicting BOO, should be performed.

## Conclusions

We confirmed the impact of abdominal pressure change in predicting endoscopically proven BOO. Our simple modification of BOOI is better correlated with endoscopically-proven obstruction than the original method. Theoretically, it is reasonable to consider the individual difference in abdominal pressure during a PFS, when determining the presence or absence of BOO. However, large prospective studies are needed to validate our suggestions.
